# COVID-19 Silver Linings - Experience of Spousal Caregivers of Persons with Dementia Engaged in Support Program

**DOI:** 10.1093/geroni/igab046.3681

**Published:** 2021-12-17

**Authors:** Andrew Nguyen, Elizabeth Stevenson, Mary Mittelman, Roscoe Nicholson, Tiffany Donley, Rebecca Salant, Steven Shirk, Maureen O'Connor

**Affiliations:** 1 Boston University School of Medicine, Boston, Massachusetts, United States; 2 NYU Langone Health, New York, New York, United States; 3 NYU Langone HealthNYU Grossman School of Medicine, NYU Grossman School of Medicine, New York, United States; 4 University of Chicago, Evanston, Illinois, United States; 5 NYU School of Medicine, New York, New York, United States; 6 NYU School of Medicine, NYU School of Medicine New York City, New York, United States; 7 Bedford Veterans Administration Medical Center, Bedford, Massachusetts, United States

## Abstract

Caring for a person with dementia (PWD) has been consistently associated with negative effects on health, including increases in caregiver depression, anxiety, and burden. Emerging studies have shown that the COVID-19 pandemic has increased these factors due to reported increases in caregiver workload and cognitive and behavioral symptoms of the PWD. We interviewed 10 spousal caregivers of PWD from the NYU Langone Alzheimer’s Disease and Related Dementias Family Support Program in Summer 2020 during the COVID-19 pandemic in order to gain feedback about their experiences during the pandemic and the transition from in-person to videoconferencing that could be used to improve services and support. Caregivers discussed the challenges faced during the pandemic but also the unique opportunities the situation presented. We report here on those positive aspects of COVID-19 from the perspective of the caregivers we interviewed. Methods: Interviews of caregivers residing with their spouses in the New York City area were conducted via videoconferencing, transcribed, deidentified, and analyzed using framework analysis methods. Results: We found that caregivers reported some positive reaction to videoconferencing that included increased support group cohesion, increased convenience, feeling less obligated to participate in events, and new opportunities for social contact. Participants also discussed positive inter-couple relationship changes such as increased quality time spent together. Our findings resonate with a body of literature focused on understanding the positive aspects of caregiving. Understanding the full presentation of the caregiver experience, including both positive and negative aspects, is important for developing interventions and resources for this unique group.

